# Comparative short-term outcomes of enhanced recovery after surgery (ERAS) program and non-ERAS traditional care in elderly patients undergoing lumbar arthrodesis: a retrospective study

**DOI:** 10.1186/s12891-021-04166-z

**Published:** 2021-03-17

**Authors:** Zhong-En Li, Shi-Bao Lu, Chao Kong, Wen-Zhi Sun, Peng Wang, Si-Tao Zhang

**Affiliations:** 1 Department of Orthopedics, Xuanwu Hospital Capital Medical University, Beijing, China; 2 National Clinical Research Center for Geriatric Diseases, Beijing, China; 3 Capital Medical University, Beijing, China

**Keywords:** Enhanced recovery after surgery, Elderly patients, Lumbar arthrodesis, Length of stay, Complication

## Abstract

**Background:**

Enhanced recovery after surgery (ERAS) program is an evidence-based improvement over non-ERAS traditional care. The aim of the present study was to analyze the safety, feasibility, and efficacy of an ERAS program in patients over 70 years undergoing lumbar arthrodesis by comparison with non-ERAS traditional care.

**Methods:**

During January 2018 to December 2018, patients enrolled received non-ERAS traditional care, while the ERAS program was implemented from January to December 2019. Demographic characteristics, comorbidities, surgical data and postoperative recovery parameters were collected from all patients. Postoperative pain scores were evaluated by visual analog scales (VAS). The clinical outcomes were length of stay (LOS), postoperative complications and postoperative pain scores. Compliance results were also collected.

**Result:**

A total of 127 patients were enrolled, including 67 patients in the non-ERAS traditional care group and 60 patients in the ERAS group. The demographic characteristics and comorbidities of the two groups showed no significant differences. The LOS of patients treated with ERAS program (13.6 ± 4.0 days) was significantly less than that of patients treated with non-ERAS traditional care (15.6 ± 3.9 days) (*p* = 0.034). Complication rate was 8.3% in the ERAS group versus 20.9% in the non-ERAS traditional care group (*p* = 0.048). VAS (back) in the ERAS group was significantly lower on postoperative day (POD) 1 and POD2. Postoperative recovery parameters were improved in the ERAS group. The overall compliance with the ERAS program was 94%.

**Conclusions:**

Based on our results, ERAS program is safer and more effective for elderly patients over 70 undergoing lumbar arthrodesis than non-ERAS traditional care.

## Background

With the advent of an aging society, the number of elderly patients with lumbar degenerative diseases requiring surgery is increasing. Lumbar arthrodesis is the most common surgical method to treat lumbar degenerative diseases [1–3]. Due to the decline of physical reserve capacity and increased comorbidities in elderly patients, the risks of perioperative complications and prolonged hospitalization need to be actively managed in clinical practice.

Enhanced recovery after surgery (ERAS) program is an evidence-based improvement over non-ERAS traditional care. Individual ERAS items are implemented during the patient’s journey through the pre-operative, intra-operative, and post-operative phases of care. Moreover, these combined factors are associated with a major shift in clinical routines, from old practices to new standardized patient care pathways [4, 5]. The ERAS program can reduce organ dysfunction and surgical stress, thereby reducing postoperative complications and length of stay (LOS) [6].

Notably, the ERAS program was first proposed based on a study in patients undergoing colon surgery. Elderly patients were the target group selected at the beginning of the program design, and the postoperative LOS was shortened to 2–3 days in patients over 80 years of age [7]. As the ERAS protocols were adopted in other surgical specialties, many factors including the characteristics of the perioperative period, the stress on the patients, and compliance rate may affect the benefit of ERAS [8–11]. Kehlet et al. recently emphasized that the ERAS program should be designed and evaluated according to specific surgery and population [12, 13]. Though the ERAS program for spinal surgery has received some attention, most of the current studies focused on minimally invasive surgery [14–18]. There are scarce data on the implementation of ERAS program in open lumbar surgery, especially in elderly patients.

The aim of the present study was to analyze the safety, feasibility, and efficacy of an ERAS program in patients over 70 years undergoing lumbar arthrodesis by comparison with non-ERAS traditional care.

## Methods

### Patients

This retrospective case-review study was conducted from January 2018 to December 2019 after the approval of the Hospital Committee and Institutional Review Board (permit data January 2018; no. 2018007) and results were reported in accordance to the STROBE statements [19]. During the first year of this study, patients enrolled received non-ERAS traditional care, while the ERAS program was implemented from January to December 2019. We prospectively collected perioperative data with analysis for the ERAS patients from medical records and database. All patients provided informed consent to the work.

The inclusion criteria included patients older than 70 years who underwent open lumbar arthrodesis with pedicle screw fixation and the main diagnosis was lumbar stenosis. Surgical treatment was performed at the following indications: (1) no improvement in clinical manifestations despite conservative treatment for a minimum of 6 months, where conservative treatment included medication, physical therapy, and up to 3 injection treatments; and (2) any pathologies requiring fusion procedure following decompression, which were degenerative spondylolisthesis, isthmic spondylolisthesis, and foraminal stenosis in the study. The exclusion criteria included (1) circumferential fusion (a combination of anterior and posterior surgery); (2) a history of previous lumbar spine surgery.

All patients underwent a standard midline posterior lumbar decompression and interbody fusion was preferred in the decompressed levels. All surgeries were performed by the same surgeon and the ERAS program was performed by the same group of trained anesthesiologists and nurses.

### Components of non-ERAS traditional care program and ERAS program

Our ERAS program was designed based on previously published pathways and the differences of details between the non-ERAS traditional care group and the ERAS group were compared in Table 1 [8, 11, 20, 21]. Non-ERAS traditional care program was performed with no unified guidelines.

**Table 1:** Components of non-ERAS Traditional care program and ERAS program

Comparisons	ERAS Program	non-ERAS Traditional care Program
**Preoperative**		
Education	Including the purpose, workflows and benefits of ERAS program, anticipated postoperative pain and expectations and risks of surgery, through verbal and handouts	Routine consultation
Nutritional counselling	Nutrition screening during the perioperative period, including nutrition screening tools and laboratory indicators. Dietitians provide personalized diet guidance and nutritional supplement to patients in need	Not standardized
Fasting	Clear fluids including carbohydrate drink allowed up to 2 h before surgery	Fasting for 10 h before surgery
Antithrombotic prophylaxis	Active/passive limb movement and antithrombotic stockings	Active/passive limb movement
Antimicrobial prophylaxis	Antibiotic prophylaxis within 1 h of incision	Antibiotic prophylaxis within 1 h of incision
**Intra-operative**		
Tranexamic acid	Used routinely	Not standardized
Standard general anesthetic protocol	Multimodal analgesia; TIVA-based anesthetic technique with propofol, lidocaine, ketamine, ketorolac, antiemetics and with up to 0.5% MAC inhaled anesthetics, avoid N2O; Depth of anesthesia monitoring	Not standardized
Avoidance of salt and water overload	Goal-directed euvolemia	Euvolemia
Maintenance of normothermia	Keeping core temperature at 36–37 °C	Not standardized
local infiltration analgesia	Used routinely	Not standardized
**Postoperative**		
Early ambulation	In-bed mobilization within 4 h after surgery; Encourage ambulation after 4 h; Early treatment with physical therapists	Recommended to rest in bed for 1–2 days
Early removal of bladder catheter	Remove the bladder catheter when returning to the ward	Remove the bladder catheter when ambulation
Early oral feeding	Oral feeding at will after recovery from anesthesia	Oral feeding on postoperative day 1
Postoperative multimodal analgesia	Adequately controlled pain; visual analog scales < 4: no analgesia or oral minimal dose of nonopioid; visual analog scales: 4–6: oral or intravenous nonopioid; visual analog scales ≥7: opioid	According to patient needs; opioid was commonly used
Stick to Discharge Criteria	Discharge Criteria: no clinical complications; Visual analog scales <3 with oral analgesics; Independent ambulation or ambulation with minimal assistance; Adequate nutrition intake	No definite discharge criteria

The perioperative multimodal analgesia (MMA) was the combination of different pain signaling pathways to improve pain control and reduce opioids.

We assigned dedicated stuff who did not participate in the study to record compliance.

### Data collection

Demographic characteristics including age, gender, body mass index (BMI), surgical level, smoker, comorbidities, American Society of Anesthesiologists (ASA) classification and preoperative Oswestry Disability Index (ODI) were collected from all patients. Surgical data including LOS, surgical time, estimated blood loss, complications and 30-day hospital readmission were recorded. Leg and back pain scores were evaluated by visual analog scales (VAS). We also recorded postoperative recovery parameters, including early ambulation, early oral feeding or early removal of catheter. The number of patients who received nutrition intervention after nutrition screening was documented. LOS was defined as the time from admission to discharge recorded in the medical record. Complications referred to all postoperative complications that occurred within 30 days after surgery. All of the complications were determined by outpatient or follow-up doctors.

### Statistics

Continuous variables were expressed as mean value ± standard deviation (Mean ± SD), and they were compared using student t test when parametric assumptions were met, otherwise, the Mann–Whitney test was performed. Statistical analysis for categorical variable was performed by the Chi-square test. Multivariate Logistic regression was used to determine association of risk factors with postoperative complications. Multivariable linear regression analysis was used to determine the association of risk factors with LOS. Differences and regressions were considered significant with p<0.05. Statistical analysis was performed using SPSS version 16.0 for Windows (SPSS Inc., Chicago, IL, USA).

## Results

### Patient demographics

A total of 127 patients were enrolled, including 67 patients in the non-ERAS traditional care group and 60 patients in the ERAS group. The demographic characteristics are detailed in Table 2. There were no statistical differences in age (*p* = 0.430), gender (*p* = 0.675), BMI (*p* = 0.769), surgical level (*p* = 0.961) between the two groups of patients. Comorbidities, ASA classification (*p* = 0.647) and preoperative ODI (*p* = 0.419) of the two groups also showed no significant differences. (Table 2).

**Table 2:** Demographic Characteristics of Patients

	ERAS	non-ERAS Traditional care	*P* value
Patients (n)	60	67	
Age (range)	73.6 ± 3.2 (70–84)	74.3 ± 4.2 (70–85)	0.430
Gender (n)			0.675
Male	22	27	
Females	38	40	
BMI	25.9 ± 3.85	26.0 ± 4.08	0.769
Surgical level			0.961
1-2levels	45	50	
≥ 3levels	15	17	
Smoker (n)	7	8	0.962
Comorbidities (n)			
Hypertension	32	36	0.964
Diabetes	19	20	0.825
Ischemic heart disease	5	8	0.503
Stroke	3	2	0.560
Arrhythmias	5	5	0.856
Gastrointestinal	2	1	0.495
Chronic lung disease	3	2	0.560
Parkinson disease	1	2	0.625
Depression	2	4	0.911
ASA classification			0.647
2	31	40	
3	23	22	
4	6	5	
Preoperative ODI (%)	59.3 ± 14.3	61.6 ± 14.7	0.419

### Clinical outcomes

The LOS of patients treated with the ERAS program (13.6 ± 4.0 days, range from 7 to 20 days) was significantly shorter than that of patients treated with non-ERAS traditional care (15.6 ± 3.9 days, range from 7 to 24 days). The surgical time in the ERAS group (176.6 ± 56.7 min) was similar to the non-ERAS traditional care group (190.4 ± 89.3 min) (*p* = 0.289). Estimated blood loss in the ERAS group (365.6 ± 207.4 ml) was similar to the non-ERAS traditional care group (337.5 ± 194.9 ml) (*p* = 0.118). Complication rate was 8.3% in the ERAS group versus 20.9% in the non-ERAS traditional care group (*p* = 0.048). After implementing ERAS program, the proportion of early ambulation increased from 7.5 to 70%. The proportion of patients with early oral feeding and removal of catheter increased respectively from 3 to 86.7% and from 14.9 to 80%. By comparison, more patients received nutrition interventions (19.4 to 45%). (Table 3).

**Table 3:** Clinical outcomes

	ERAS (*n* = 60)	non-ERAS Traditional care (*n* = 67)	*P* value
LOS (range), days	13.6 ± 4.0 (7–20)	15.6 ± 3.9 (7–24)	0.034
Surgical time, min	186.6 ± 56.7	190.4 ± 89.3	0.289
Estimated blood loss, ml	337.5 ± 194.9	365.6 ± 207.4	0.118
Complications, n (rate)			0.048
No	55 (91.7)	53 (79.1)	
Yes	5 (8.3)	14 (20.9)	
Early ambulation, n (rate)			<0.0001
No	18 (30.0)	62 (92.5)	
Yes	42 (70.0)	5 (7.5)	
Early oral feeding, n (rate)			<0.0001
No	8 (13.3)	65 (97.0)	
Yes	52 (86.7)	2 (3.0)	
Early removal of catheter, n (rate)			<0.0001
No	12 (20.0)	57(85.1)	
Yes	48 (80.0)	10 (14.9)	
Nutritional intervention, n (rate)			0.002
No	33 (55.0)	54 (80.6)	
Yes	27 (45.0)	13 (19.4)	

Complications are summarized in Table 4. Among the 5 patients with complications in the ERAS group, two had superficial infection, one suffered from cerebrospinal fluid leakage, one suffered from electrolyte abnormality and one experienced arrhythmia. Complications in the non-ERAS traditional care group were distributed as follows: urinary tract infection, partial root injuries, arrhythmia, deep vein thrombosis and deep wound infection, each in one case, two patients suffered from superficial infection, three patients suffered from electrolyte abnormality, and four patients experienced delirium. There were no complications requiring readmission within 30 days of surgery in both groups.

**Table 4:** List of complications of the two groups

Complications	ERAS	Non-ERAS traditional care
Total	5	14
Superficial infection	2	2
Electrolyte abnormality	1	3
Arrhythmia	1	1
Urinary tract infection	0	1
Deep vein thrombosis	0	1
Deep wound infection	0	1
Delirium	0	4
Surgical complications		
Cerebrospinal fluid leakage	1	0
Partial root injuries	0	1

Average VAS (back) in the ERAS group was significantly lower than that in the non-ERAS traditional care group on postoperative day (POD) 1 (3.8 ± 1.7 versus 5.7 ± 2.3, *p* = 0.028) and POD2(3.6 ± 1.9 versus 4.5 ± 2.2, *p* = 0.043). Whereas, there were no statistically significant differences between the groups on POD3 (3.1 ± 1.2 versus 3.8 ± 1.7, *p* = 0.122) and POD4 (2.7 ± 0.5 versus 3.2 ± 0.9, *p* = 0.363). There were no differences in the VAS (leg) postoperatively. (Table 5).

**Table 5:** The comparisons of average VAS between the two groups on POD1–4

VAS (back)				VAS (leg)		
comparison	ERAS	non-ERAS Traditional care	*P* value	ERAS	non-ERAS Traditional care	*P* value
POD 1	3.8 ± 1.7	5.7 ± 2.3	*p* = 0.028	3.2 ± 1.5	3.8 ± 1.7	*p* = 0.137
POD 2	3.6 ± 1.9	4.5 ± 2.2	*p* = 0.043	2.5 ± 1.6	3.5 ± 1.2	*p* = 0.088
POD 3	3.1 ± 1.2	3.8 ± 1.7	*p* = 0.122	2.0 ± 1.3	2.5 ± 0.9	*p* = 0.230
POD 4	2.7 ± 0.5	3.2 ± 0.9	*p* = 0.363	1.8 ± 0.8	2.0 ± 1.0	*p* = 0.594

### Multivariable analyses

Multivariable linear regression analysis was performed to determine the association of various factors with LOS. Implementation of the non-ERAS traditional care program (*p* = 0.006) and higher preoperative ODI (*p* = 0.012) were correlated with prolonged LOS. On the other hand, age (*p* = 0.579), BMI (*p* = 0.351), surgical level ≥ 3 (*p* = 0.083) and surgical time (*p* = 0.127) were not related to LOS (Table 6).

**Table 6:** Multivariable linear regression for LOS

Characteristic	Multivariable linear regression for LOS	
	Coefficient (95% CI)	*p* value
Age	0.15(−0.09 to 0.32)	0.579
BMI	−0.37(−0.90 to 1.12)	0.351
Surgical level ≥ 3	1.94(−0.76 to 3.16)	0.083
ASA ≥ 3	1.15(−0.42 to 2.53)	0.238
Surgical time	2.27(−1.2 to 3.34)	0.127
ERAS	−3.08(−5.12 to −1.14)	0.006
Preoperative ODI (%)	0.94(0.25 to 2.04)	0.012

Multivariable logistic regression showed that implementation of ERAS program (*p* = 0.040) was associated with decrease in complications. The other characteristics were not associated with complications. (Table 7).

**Table 7:** Multivariable logistic regression for any complications

Characteristic	Multivariable logistic regression for any complications	
	OR (95% CI)	*p* value
Age	1.02(0.97–1.30)	0.248
BMI	0.94(0.86–1.21)	0.062
Surgical level ≥ 3	1.87(0.77–4.50)	0.167
ASA ≥ 3	2.06(0.72–5.91)	0.203
Surgical time	2.23(0.87–3.86)	0.337
ERAS	0.58(0.19–0.93)	0.040
Preoperative ODI (%)	0.91(0.87–1.64)	0.320

### Compliance with the ERAS program

Compliance with the ERAS program is illustrated in Table 8. In general, high compliance rates were achieved for the pre-operative and Intra-operative ERAS items. In contrast, compliance of the postoperative ERAS items was relatively low: early ambulation (70%), early removal of bladder catheter (86.7%), early oral feeding (80%), stick to discharge criteria (78.3%). The overall compliance was 94%.

**Table 8:** Compliance with the ERAS program

Variable	n (%)
Pre-operative ERAS items	
Patient education	60 (100)
Nutritional counselling	60 (100)
No prolonged fasting	60 (100)
Fluid and carbohydrate loading	59 (98.3)
Antithrombotic stockings	58 (96.7)
Antimicrobial prophylaxis	60 (100)
Intra-operative ERAS items	
Tranexamic acid	60 (100)
Avoidance of salt and water overload	60 (100)
Maintenance of normothermia	60 (100)
local infiltration analgesia	60 (100)
Postoperative ERAS items	
Early ambulation	42 (70.0)
Early removal of bladder catheter	52 (86.7)
Early oral feeding	48 (80.0)
Stick to discharge criteria	47 (78.3)
Perioperative multimodal analgesia	60 (100)
Overall compliance (rate)	94.0

## Discussion

To the best of our knowledge, this is the first study focusing on the implementation of ERAS program in elderly patients undergoing lumbar arthrodesis. Similar to the results of ERAS program in minimal invasive lumbar surgery [15, 16], this study found that the ERAS program significantly reduced the incidence of complications and LOS, and the majority of elderly patients could complete the pathway. The standardized multimodal analgesia significantly reduced postoperative pain levels in the ERAS group. Only patients undergoing lumbar arthrodesis were included in this study to avoid the bias caused by surgical types.

Due to the influence of non-clinical factors such as culture, doctor-patient relationship, and insurance system, although the LOS of patients in the ERAS group was significantly shortened in our study, it was still longer than that in other studies [5, 17, 18]. It is reported that elderly patients undergoing lumbar spine surgery have extended LOS, because patients with advanced age are more likely to suffered from more baseline comorbidities and postoperative complications that may require further medical or surgical intervention [22, 23]. Moreover, elderly patients commonly experienced issues of decreased ability to perform daily activities and difficulty with self-care, which leads to reluctance to discharge, even if the criteria are met [24]. In our study, the LOS was prolonged to 24 days of 1 patient in the non-ERAS traditional care group because of deep wound infection. The patient had to be taken to the operating room for wound washout. Another patient in the non-ERAS traditional care group delayed LOS to 22 days because of postoperative delirium. In our ERAS group, 1 patient experienced delayed discharge (LOS = 20 days) for nonsurgical superficial infection. Our study also showed that higher preoperative ODI was associated with prolonged LOS, because worse preoperative motor capacity usually leads to longer time for first ambulation. The implementation of ERAS can reduce occurrence of complications, and provide adequate pain control, which are important components of our discharge criteria.

Similar to previous studies [25–27], our results showed that the implementation of ERAS program was associated with lower complications. We believe that there are several factors in the study contributing to the decrease in complications. Early removal of bladder catheter and standard antimicrobial prophylaxis reduce the risk of infectious complications such as urinary tract infection and wound infection [28–30]. Thrombosis-related complications can be decreased by active/passive limb movement, antithrombotic stockings and early ambulation. Notably, advanced age is one of the main risk factors for postoperative delirium, and the incidence of postoperative delirium after elective lumbar surgery can be as high as 15%, which can lead to nursing difficulties and lower compliance with the ERAS protocols [31–33]. Postoperative delirium has rarely been discussed in ERAS studies, but it is extremely important for the prognosis of patients, especially in elderly patients [12, 34]. In this study, no patients in the ERAS group suffered from postoperative delirium in contrast to 4 cases in non-ERAS traditional care group. Early recovery of normal life, multimodal analgesia and depth of anesthesia monitoring in the ERAS program can effectively reduce surgical stress and the risk of delirium [31]. Opioids are considered to be the cornerstone of analgesics for severe pain, but opioid abuse increases the risk of postoperative delirium [35].

Improved pain control has been proved to be correlated with decreased risks of wound healing and Infectious complications, delirium, delayed mobilization, and prolonged LOS [36, 37]. Multimodal analgesia was applied in our ERAS program, and a standardized analgesic strategy was established based on patient-reported pain VAS score. Significantly lower back pain scores on POD 1–2 and shortened LOS suggested improved pain control in the ERAS program. And nonopioid-preferable pain management can reduce opioid side effects and long-term dependence. It is necessary to weigh the side effects against the strong potency of opioids. Although some guidelines and reviews mentioned about multimodal analgesia in ERAS program, there are still some controversies, such as the application of patient-specific multimodal analgesia programs for elderly patients and multimodal analgesic management for pre-operative opioid users [12, 35, 38, 39].

Preoperative education helps elderly patients gain a clear understanding of the expectations of surgeries and build confidence in perioperative recovery [40]. Due to the decline in visual and auditory functions of elderly patients, the education was through verbal and handouts, with an emphasis of involvement of family members. Understanding the patient’s expectations, preferences and the burden of postoperative care can help medical teams determine better treatment options to truly improve quality of life.

A growing number of studies have recognized that malnutrition can lead to adverse outcomes of spinal surgery, especially for elderly patients [41–44]. Increased risk of malnutrition in aging population is due to living alone, chronic diseases and poor dietary habits [45]. Dietitians participated in daily rounds and identified the patients who were malnourished or at risk of malnutrition through nutrition screening tools and laboratory tests. Personalized diet guidance and nutritional supplement were provided to patients in need. Unlike other studies [46], economic factors and medical insurance system were taken into consideration and no additional nutritional supplements were provided for elderly patients with good nutritional status, but instead professional guidance on perioperative diets was given. Our results showed a significant increase in the proportion of patients receiving nutritional supplements in the ERAS group, indicating that previous malnutrition or risks in the non-ERAS traditional care group may be ignored or not intervened.

The neglect of compliance leads to doubts about the impact of ERAS program on the prognosis. Our results illustrated that the overall ERAS compliance was as high as 94%, and the compliance of preoperative and intraoperative items was better than postoperative items. We considered that the postoperative ERAS procedures are affected by the patient’s subjective consciousness and the actual condition, while the preoperative and intraoperative steps depend more on the executive capability of the medical team. The close and timely communication of the ERAS team helps to identify potential difficulties and optimize ERAS procedures. Recent studies have shown that continual auditing of the protocol can help to improve compliance [38].

Although the compliance of early ambulation was only 70% in our study, the overall time until ambulation postoperatively was greatly shortened. Previous studies have shown that early ambulation is associated with decreased morbidity and adverse events after elective lumbar spine surgery [47]. However, preoperative deterioration of motor function, endurance and coordination makes early ambulation more difficult. And early ambulation is often accompanied by orthostatic intolerance, such as dizziness and nausea, which increases the risk of aspiration and fall [47, 48]. Therefore, for elderly patients, early ambulation should be encouraged rather than enforced, and should be accompanied by the presence of professional caregivers and patient confirmation of no obvious discomfort after sitting up.

In this study, 86.7% of patients complied with early removal of bladder catheter. While early removal of bladder catheter may increase the risk of reinsertion and urinary retention [30, 49]. It significantly reduces the risk of urinary tract infections and gives patients confidence to return to normal life, which is helpful in shortening LOS [50]. Patient’s urination should be closely monitored after early removal of the bladder catheter. Prudence should be taken to determine whether reinsertion is required if there is a possibility of urine retention. Prolonged bladder catheter carrying may be justified for elderly patients with prostate disease.

Although we developed detailed discharge criteria, the compliance was only 78.3%. Geriatric syndromes (such as constipation, incontinence or pressure sores) may cause the elderly not to be discharged even if the discharge criteria are met. In addition, due to inadequate conditions in community medical care facilities and nursing homes, inconvenience of life after discharge and concerns about readmission, some of the elderly patients were reluctant to be discharged [9, 12, 51]. Therefore, we should proactively address the psychosocial problems that the elderly may encounter. Detailed guidance on comorbidities and fostering trust between patients and medical teams can help patients relieve their anxieties. Rehabilitation guidance and telephone follow-up allow patients to be discharged safely.

Individual ERAS components were not independently linked to the prognosis indicators. Nontheless, the ERAS program is a multimodal pathway and all elements have an additive effect on prognosis. The ERAS program is based on “first better, then faster”, and extension of LOS in elderly patients with special comorbidities should be granted on a case by case basis.

### Limitations

This was a single-center prospective comparative study with a relatively small sample size. The ERAS and non-ERAS traditional care groups were in different time frames, which may cause bias in the analysis. It is not possible to assess the impact of ERAS program on cost savings because no cost information was collected. Due to the short follow-up period, we were unable to evaluate the effects of ERAS program on long-term complications and functional recovery.

## Conclusion

Based on our short-term results, the ERAS program is safer and more effective for elderly patients over 70 undergoing lumbar arthrodesis than non-ERAS traditional care in perioperative period. The ERAS program significantly reduced the incidence of complications and LOS. Due to the characteristics of elderly patients, we should pay attention to compliance when implementing ERAS. For perioperative safety, we should appropriately allow the extension of LOS in elderly patients with special comorbidities.

## Data Availability

Request for datasets generated and analyzed during the current study can be addressed to the corresponding author.

## Abbreviations

ERAS: Enhanced recovery after surgery
ODI: The Oswestry Disability Index
VAS: Visual analog scales
LOS: Length of stay
ASA: American Society of Anesthesiologists
POD: Postoperative day
